# The Yeast Complex I Equivalent NADH Dehydrogenase Rescues *pink1* Mutants

**DOI:** 10.1371/journal.pgen.1002456

**Published:** 2012-01-05

**Authors:** Sven Vilain, Giovanni Esposito, Dominik Haddad, Onno Schaap, Mariya P. Dobreva, Melissa Vos, Stefanie Van Meensel, Vanessa A. Morais, Bart De Strooper, Patrik Verstreken

**Affiliations:** 1 VIB Center for Biology of Disease, Katholieke Universiteit Leuven, Leuven, Belgium; 2 Center for Human Genetics and Leuven Research Institute for Neurodegenerative Diseases (LIND), Katholieke Universiteit Leuven, Leuven, Belgium; Max Planck Institute for Biology of Aging, Germany

## Abstract

Pink1 is a mitochondrial kinase involved in Parkinson's disease, and loss of Pink1 function affects mitochondrial morphology via a pathway involving Parkin and components of the mitochondrial remodeling machinery. Pink1 loss also affects the enzymatic activity of isolated Complex I of the electron transport chain (ETC); however, the primary defect in *pink1* mutants is unclear. We tested the hypothesis that ETC deficiency is upstream of other *pink1*-associated phenotypes. We expressed *Saccaromyces cerevisiae* Ndi1p, an enzyme that bypasses ETC Complex I, or sea squirt *Ciona intestinalis* AOX, an enzyme that bypasses ETC Complex III and IV, in *pink1* mutant *Drosophila* and find that expression of Ndi1p, but not of AOX, rescues *pink1*-associated defects. Likewise, loss of function of subunits that encode for Complex I–associated proteins displays many of the *pink1*-associated phenotypes, and these defects are rescued by Ndi1p expression. Conversely, expression of Ndi1p fails to rescue any of the *parkin* mutant phenotypes. Additionally, unlike *pink1* mutants, fly *parkin* mutants do not show reduced enzymatic activity of Complex I, indicating that Ndi1p acts downstream or parallel to Pink1, but upstream or independent of Parkin. Furthermore, while increasing mitochondrial fission or decreasing mitochondrial fusion rescues mitochondrial morphological defects in *pink1* mutants, these manipulations fail to significantly rescue the reduced enzymatic activity of Complex I, indicating that functional defects observed at the level of Complex I enzymatic activity in *pink1* mutant mitochondria do not arise from morphological defects. Our data indicate a central role for Complex I dysfunction in *pink1*-associated defects, and our genetic analyses with heterologous ETC enzymes suggest that Ndi1p-dependent NADH dehydrogenase activity largely acts downstream of, or in parallel to, Pink1 but upstream of Parkin and mitochondrial remodeling.

## Author Summary

Parkinson's disease is the most common neurodegenerative movement disorder, and mutations in several genes are known to cause the disorder. A common theme among several PD–associated genes is a link to mitochondria, organelles that use their electron transport chain to generate ATP. One of the PD–associated genes encodes a mitochondrial kinase Pink1, but it is not known what the primary role of Pink1 is within the mitochondria. Indeed, loss of Pink1 function in cells and model organisms results not only in mitochondrial morphological defects but also in an enzymatic deficit at the level of the first protein complex in the electron transport chain. Here, we express yeast Ndi1p (an enzyme that can bypass electron transport in Complex I) and sea squirt alternative oxidase (an enzyme that can bypass electron transport in Complex III/IV) in *pink1* mutant fruit flies and find that supplementing the mutants with Ndi1p, but not with alternative oxidase, results in significant rescue of multiple phenotypes. Conversely, mitochondrial morphological defects in *pink1* mutants are rescued by genetically improving mitochondrial fission, but this manipulation fails to improve the enzymatic deficiency at the level of Complex I. Our data thus pinpoint an important mode of action of Pink1 at the level of Complex I, and this action at least in part precedes defects at the level of mitochondrial remodeling.

## Introduction

Parkinson's disease (PD (OMIM #168600)) is the most common neurodegenerative movement disorder [1]. While diverse processes including autophagy, apoptosis, oxidative stress and accumulation of protein inclusions have been implicated in the etiology of the disease, mitochondrial dysfunction appears to play a central role as well [2]–[4]. Mitochondrial toxins, such as MPTP (1-methyl-4-phenyl-1,2,3,6-tetrahydropyridine) or rotenone, that block Complex I of the mitochondrial electron transport chain (ETC) cause clinical features reminiscent of PD in humans and are commonly used to create animal models of the disease [5], [6]. Furthermore, Complex I deficiency is often observed in neurons of PD patients [7] and mutations in genes causing familial forms of PD, including *pink1* (PARK6, OMIM #605909, Gene ID: 65018), *parkin* (PARK2, OMIM #600116, Gene ID: 5071) and *DJ-1* (PARK7, OMIM #606324, Gene ID: 11315) result in defects in mitochondrial morphology and/or function in model organisms [8]–[14]. Molecular genetic analyses of PD-associated genes will thus yield important insights into the mechanisms of PD.

Pink1 (CG4523 Gene ID: 31607) is a serine/threonine kinase involved in maintaining mitochondrial integrity [8]–[10] and loss-of-function mutants show hallmark mitochondrial defects including male sterility, an inability of most flies to fly as well as an inability to maintain synaptic transmission during intense stimulation, a deficit that can be rescued by supplementing synapses with ATP [15], [16]. The observation of larger clumped mitochondria in *pink1* mutants suggests a model where Pink1 is involved in the clearance of dysfunctional mitochondria [17]–[21]. This is in line with experiments that show an alleviation of *pink1*-associated phenotypes by over-expression of Parkin (CG10523 Gene ID: 40336), an E3 ubiquitin ligase involved in mitophagy [8]–[10] and by the notion that loss-of-function *pink1* trumps Parkin recruitment to mitochondria [18]. Furthermore, the morphological defects in *pink1* mutants can be modulated by altering the levels of proteins involved in mitochondrial fusion or fission, and this is thought to facilitate mitophagy [17], [19], [21], [22]. However, these studies are not conclusive as functional defects in *pink1* mitochondria at the level of Complex I have been observed in the absence of severe morphological alterations [15], [23] and such functional defects can eventually result in mitochondrial morphological alterations [24]–[26]. These results suggest an alternative model where functional defects in *pink1* mutant mitochondria precede morphological alterations and mitophagy.

To determine if Pink1 acts to regulate ETC function we performed genetic studies with heterologous alternative enzymes that can bypass either Complex I, or Complex III and IV. Although each of the ETC complexes in flies (and humans) comprise numerous proteins (Complex I contains more than 40 subunits in humans and in flies), *Saccharomyces cerevisiae* Ndi1p (Gene ID: 854919) (*UAS-NDI1*) constitutes an alternative NADH oxidoreductase that transfers electrons from NADH to ubiquinone, delivering electrons to downstream complexes and therefore can be used to bypass electron transport in Complex I in higher order species [27], [28]. Similarly, ‘*Ciona intestinalis*’ alternative oxidase AOX (Gene ID: 3293227) is able to bypass the cytochrome *c* chain and Complexes III and IV by using electrons from ubiquinol to reduce oxygen [29]. While neither Ndi1p, nor AOX themselves transfer protons across the inner mitochondrial membrane, they may add to the proton motive force by facilitating the ubiquinone cycle, thus contributing to Complex III and IV mediated proton translocation upon expression of Ndi1p, or to Complex I mediated proton translocation upon expression of AOX. Illustrating this idea, expression of Ndi1p in *Drosophila* can rescue partial loss-of-function mutations in Complex I components and confers rotenone resistance [30], [31], and expression of AOX can rescue partial loss-of-function mutations in Complex III and IV components and confers cyanide resistance [32]. Hence, Ndi1p and AOX allow us to genetically dissect the ETC.

Here, we assay the role of Complex I in *pink1*-associated defects and find that Ndi1p can rescue *pink1* phenotypes while AOX cannot. Similarly, loss-of-function of a Complex I component phenocopies many of the *pink1* mutant phenotypes and these defects can also be rescued by expression of Ndi1p. In contrast, expression of Ndi1p fails to rescue *parkin* mutants, indicating that Ndi1p acts downstream or in parallel to Pink1 but upstream of Parkin. Further supporting this model, we do not find reduced enzymatic activity of Complex I in *parkin* mutants, and while modulating mitochondrial remodeling using Drp1 (CG3210 Gene ID: 33445) or Opa1 (CG8479 Gene ID: 36578) can rescue the defects in mitochondrial morphology in *pink1* mutants, these manipulations do not rescue decreased enzymatic activity of Complex I observed in *pink1* mutants. Thus, our studies suggest that the defects at Complex I in *pink1* mutants are upstream of several of the events that lead to *pink1*-associated phenotypes.

## Results

### Yeast Ndi1p rescues *pink1*

**Figure 1:**
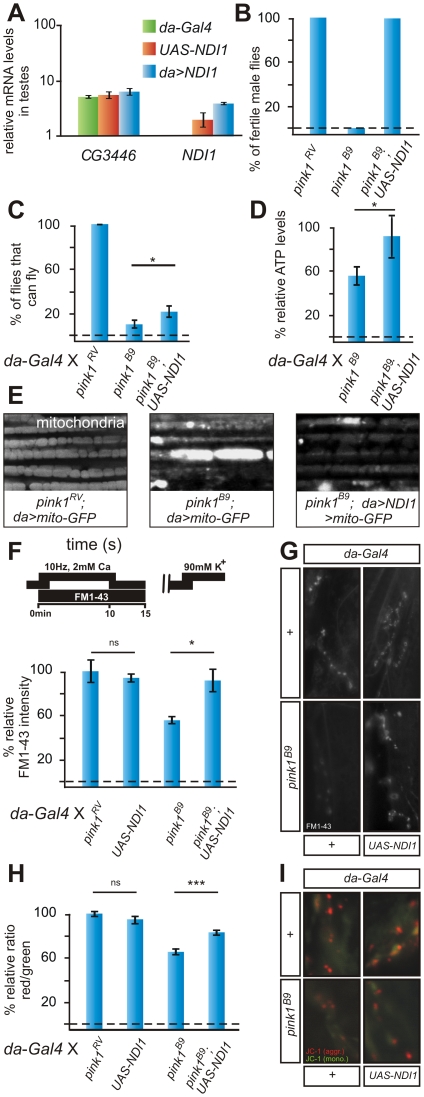
*NDI1* transgene is expressed and rescues *pink1* mutant phenotypes. (A) Quantitative RT-PCR using primers to *CG3446*, a component of ETC Complex I and to *NDI1*, in testes using tissue of the following genotypes *w*; *da-Gal4* (green)/*+*, *w*; *UAS-NDI1* (orange) and *w*; *UAS-NDI1*/*+*; *da-Gal4*/+ (blue). Data normalized to housekeeping genes (see Text S1). (B) Quantification of fertility of *w pink1^RV^* control male flies of *w pink1^B9^*and of *w pink1^B9^*; *UAS-NDI1* male flies. “RV”: wild type *pink1* gene (precise *P* element excision); “B9”: mutant *pink1^B9^* allele (n = 30 animals). (C) Quantification of flight in control (*w pink1^RV^*; *da-Gal4*/*+*) in *pink1^B9^* mutant (*w pink1^B9^*; *da-Gal4*/*+*) and in *pink1^B9^* mutant flies that express Ndi1p (*w pink1^B9^*; *UAS-NDI1*/*+*; *da-Gal4*/*+*). Student's t-test: * *p*<0.05. Error bars SEM, n = 10 experiments with 5 flies each. (D) Relative ATP levels of *pink1^B9^* mutant flies (*w pink1^B9^*; *da-Gal4/+*) normalized to *pink1^RV^* control flies (*w pink1^RV^*; *da-Gal4/+*) and of *pink1^B9^* mutant flies that express Ndi1p (*w pink1^B9^*; *UAS-NDI1/+*; *da-Gal4/+*) normalized to control flies that express Ndi1p (*w*; *UAS-NDI1/+*; *da-Gal4/+*). Student's t-test: * *p*<0.05. Error bars SEM, n = 3 experiments with 5 flies each. (E) GFP labeling of mitochondria from the adult indirect flight muscles of the following genotypes *w pink1^RV^*; *UAS-mito:GFP*/*+*; *da-Gal4*/+ and *w pink1^B9^*; *UAS-mito:GFP*/+; *da-Gal4*/+ and *w pink1^B9^*; *UAS-mito:GFP*/*UAS-NDI1*; *da-Gal4*/*+*. (F,G) Quantification (F) and labeling (G) of Reserve Pool (RP) vesicles in *pink1^RV^* controls (*w pink1^RV^*; *da-Gal4/+*) in larvae expressing the *NDI1* transgene (*w*; *UAS-NDI1/+*; *da-Gal4/+*), in *pink1^B9^* mutants (*w pink1^B9^*; *da-Gal4*/+) and in *pink1^B9^* mutants expressing Ndi1p (*w pink1^B9^*;*UAS-NDI1*/*+*; *da-Gal4*/*+*), using the stimulation protocol shown on the left (methods). Student's t-test: * *p*<0.05; ns, not significant. Error bars SEM, n>8 synapses from 4 animals. (H,I) Quantification of red to green JC-1 fluorescence (H) and examples (I) at NMJ boutons in larvae of the genotypes indicated in (F–G). Student's t-test: *** *p*<0.0001; ns, not significant. Error bars SEM, n>16 synapses from 4 animals.

Electron transfer activity of the mitochondrial multi-protein Complex I, NADH:ubiquinone oxidoreductase (EC 1.6.5.3) can be recapitulated by a single yeast protein, Ndi1p [31], [33], [34]. To determine whether the reduced respiratory chain activity in *pink1* mutants reported previously [15] are an upstream defect in the mutants, we generated transgenic flies that harbor a UAS-*Sacharomyces cerevisiae NDI1* construct allowing expression under the control of GAL4. Quantitative RT-PCR using RNA from flies that harbor the *UAS-NDI1* transgene and the ubiquitously expressing *da-GAL4* driver indicates that *NDI1* expression levels in such animals is similar to that of an endogenously expressed Complex I component *CG3446* (Figure S1A). In addition, quantitative RT-PCR using RNA of UAS-*NDI1* bearing flies, in the absence of GAL4 also shows low but significant expression of *NDI1* RNA, particularly in the male reproductive organ (Figure 1A, blue). Ndi1p confers rotenone insensitive NADH oxidoreductase activity in flies as the enzymatic activity of isolated Complex I in the presence of rotenone, a Complex I inhibitor, remains high in mitochondria from flies expressing Ndi1p, but is dramatically reduced in rotenone-treated mitochondria from control flies (Figure S1B) [30], [31]. Ndi1p expression in *Drosophila* is benign as ubiquitous expression (*da-GAL4*) does not lead to obvious behavioral or developmental abnormalities (Figure S1C, S1D). Ndi1p expression rescues lethality associated with RNAi-induced systemic loss-of-function of *CG18624* (Gene ID: 31697), an evolutionary conserved Complex I component, and also the mitochondrial defects associated with loss of *CG12079* (Gene ID: 38378), another conserved Complex I component (below). These data confirm that Ndi1p is functional.

Next, we generated *pink1* mutant animals that express Ndi1p. While *pink1* mutant males are sterile, low expression of Ndi1p is sufficient to completely revert this defect (Figure 1B). Given that *pink1* is located on the X-chromosome, homozygous *pink1* females are never observed. However, in the presence of *NDI1*, fertile homozygous *pink1* female flies are obtained (Figure S2A), whose genetic makeup we confirmed by genomic PCR (Figure S2B). Furthermore, while in *pink1* mutants morphological defects in spermatid mitochondria are apparent [10], [31], expression of Ndi1p also rescues these mitochondrial defects (data not shown). Thus, expression of Ndi1p rescues *pink1*-associated male sterility and mitochondrial morphological defects in the germline to a level indistinguishable from wild type controls.

*Drosophila* flight muscles require large amounts of metabolic energy supplied by mitochondria. In adult *pink1* mutant flies, mitochondrial deficits lead to muscle degeneration and a severe defect to fly. While expression of Ndi1p using *da-GAL4* does not affect flight (Figure S1C), expression in *pink1* mutants improves flight (Figure 1C). Although expression of Ndi1p does not restore the *pink1* flight defect to control levels, it is important to note that previous experiments demonstrating rescue of *Pink1* phenotypes using over-expressed Parkin showed very similar results [9].

Ndi1p expression also rescues degeneration of the indirect flight muscles in *pink1* mutants as evaluated by decreased indentations in the thoraces of the flies (Figure S2C). As flight muscle degeneration correlates with an accumulation of enlarged mitochondria in *pink1* mutants [8]–, we labeled the mitochondrial pool using mito-GFP (Figure 1E). In adult *pink1* flight muscles, the mitochondria appear enlarged and clumped when compared to controls (Figure 1E, middle) and Ndi1p expression in *pink1* mutants partially trumps this defect (Figure 1E, right). Finally, *pink1* mutants that express Ndi1p have an increase in ATP levels compared to *pink1* mutants not expressing Ndi1p (Figure 1D). Thus, enhanced ATP levels in *pink1* mutants that express Ndi1p may dampen mitochondrial dysfunction and diminish muscle degeneration.

Previous data indicated that mitochondrial defects cause synaptic vesicle trafficking and neurotransmitter release defects at *pink1* mutant synapses [15], [23]. We therefore measured neurotransmitter release at the *Drosophila* third instar neuromuscular junction (NMJ). Upon stimulation of the motor neuron at 1 Hz, *pink1* mutants, *pink1* mutants that express Ndi1p, as well as control animals that express Ndi1p, show normal neurotransmitter release as gauged by the amplitude of the excitatory junctional potential (EJP) (Figure S3A). In contrast, when stimulated at high frequency (10 Hz), neurotransmitter release in *pink1* mutants gradually declines, in line with a defect to mobilize ‘reserve pool’ (RP, see below) vesicles that are only used under such ‘stressed’ conditions [16], [35]. Interestingly, *pink1* mutants that express Ndi1p maintain normal levels of synaptic transmission during a 10 Hz 10 min stimulation paradigm (Figure S3B). These data indicate that synaptic transmission defects at *pink1* mutant NMJs are also rescued by expression of Ndi1p. As previously shown, this neurotransmission deficit is likely caused by a lack of mobilization of the reserve pool synaptic vesicles within NMJ boutons [15]. We used the fluorescent dye FM 1–43 to label vesicles loaded in the RP [36] using the stimulation paradigm depicted in Figure 1F [37]. While *pink1* mutants show a significant reduction in RP vesicle labeling, *pink1* mutants that express Ndi1p display labeling of RP vesicles very similar to controls (Figure 1F, 1G). Thus, synaptic function deficits in *pink1* mutants are alleviated by expression of yeast Ndi1p.

In *pink1* mutant motor neurons, mitochondria are morphologically normal but show only partial mitochondrial membrane depolarization [15]. We assessed the mitochondrial membrane potential in *pink1* mutants rescued with Ndi1p using the ratiometric dye JC-1. [16], [38]. Interestingly, compared to *pink1* mutants, mitochondria at synaptic boutons of *pink1* mutants that express Ndi1p are significantly more polarized and show more intense red JC-1 labeling (Figure 1H, 1I). Although Ndi1p itself is not involved in proton transfer over the inner mitochondrial membrane, our data indicate that expression of Ndi1p can restore a negative mitochondrial membrane potential in *pink1* mutants. Potentially Ndi1p improves electron transfer from NADH to complex III/IV that in turn helps to restore the proton gradient in mitochondria at synapses.

### Expression of AOX does not rescue *pink1* mutant phenotypes

**Figure 2:**
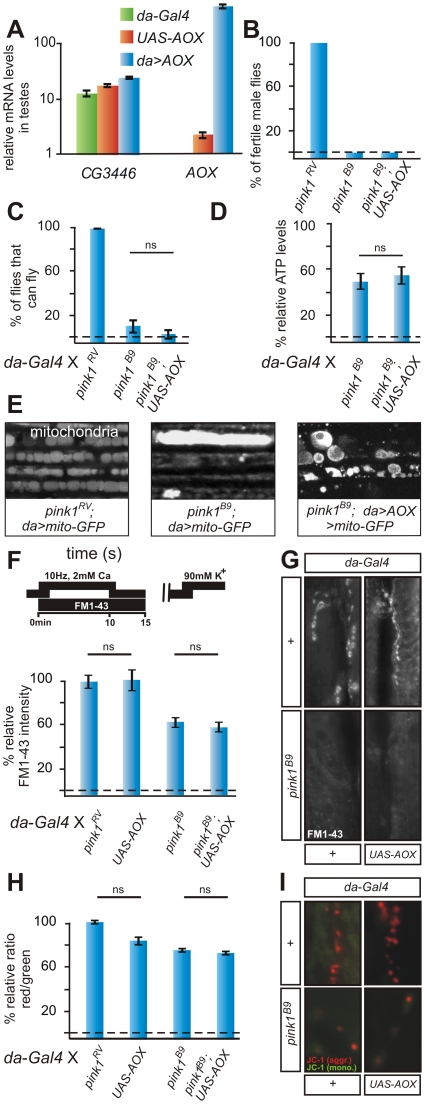
*AOX* does not rescue *pink1* mutants. (A) Quantitative RT-PCR using primers to *CG3446* and to *AOX*, in testes using tissue of the following genotypes *w*; *da-Gal4*/*+* (green), *w*; *UAS-AOX* (orange) and *w*; *UAS-AOX*/*+*; *da-Gal4*/+ (blue). Data normalized to housekeeping genes (see Text S1). (B) Quantification of fertility of *w pink1^RV^* control male flies of *pink1^B9^*and of *w pink1^B9^*; *UAS-AOX* male flies (n = 20 animals). (C) Quantification of flight in control (*w pink1^RV^*; *da-Gal4*/*+*) in *pink1^B9^* mutant (*w pink1^B9^*; *da-Gal4*/*+*) and in *pink1^B9^* mutant flies that express *AOX* (*w pink1^B9^*; *UAS-AOX*/+; *da-Gal4*/*+*). Student's t-test: ns, not significant. Error bars SEM, n = 6 experiments with 5 flies each. (D) Relative ATP levels of *pink1^B9^* mutant flies (*w pink1^B9^*; *da-Gal4/+*) normalized to *pink1^RV^* control flies (*w pink1^RV^*; *da-Gal4/+*) and of *pink1^B9^* mutant flies that express AOX (*w pink1^B9^*; *UAS-AOX/+*; *da-Gal4/+*) normalized to control flies that express AOX (*w*; *UAS-AOX/+*; *da-Gal4/+*). Student's t-test: ns, not significant. Error bars SEM, n = 3 experiments with 5 flies each. (E) GFP labeling of mitochondria from the adult indirect flight muscles of the following genotypes *w pink1^RV^*; *UAS-mito:GFP*/*+*; *da-Gal4*/+ and *w pink1^B9^*; *UAS-mito:GFP*/+; *da-Gal4*/+ and *w pink1^RV^*; *UAS-mito:GFP*/*UAS-AOX*; *da-Gal4*/+. (F,G) Quantification (F) and labeling (G) of Reserve Pool (RP) vesicles in *pink1^RV^* controls (*w pink1^RV^*; *da-Gal4/+*) in larvae expressing the AOX transgene (*w*; *UAS-AOX/+*; *da-Gal4/+*), in *pink1^B9^* mutants (*w pink1^B9^*; *da-Gal4/+*) and in *pink1^B9^* mutants expressing AOX (*w pink1^B9^*; *UAS-AOX/+*; *da-Gal4/+*), using the stimulation protocol shown on the left. Student's t-test: * *p*<0.05; ns, not significant. Error bars SEM, n>8 synapses from 4 animals. (H,I) Quantification of red to green JC-1 fluorescence (H) and examples (I) at NMJ boutons in larvae of the genotypes indicated in (F–G). Student's t-test: ns, not significant. Error bars SEM, n>16 synapses from 4 animals.

To further test the specificity of Ndi1p-dependent rescue of *pink1*-associated phenotypes, we also tested the ability of *Ciona intestinalis* AOX to bypass *pink1* defects. Our rationale is that AOX can also transport electrons but at a different site within the ETC: AOX uses electrons from ubiquinol to reduce oxygen and thereby bypasses both Complex III and IV [29], [32]. We confirm the functionality of AOX in flies because lethality induced by expression of RNAi to *cyclope* (CG14028 Gene ID 46040) that encodes a Complex IV component, is rescued upon expression of AOX (data not shown) [32]. Similar to *NDI1* we also find basal expression of *AOX* in the male reproductive organ that is much induced by the presence of *da-GAL4* (Figure 2A) and ubiquitous expression of AOX also does not affect flight, muscle degeneration or the mitochondrial membrane potential (Figure S4A, S4B; see below). In contrast to Ndi1p, expression of AOX completely fails to alleviate *pink1*-associated phenotypes such as male fertility, flight or mitochondrial morphology (Figure 2B, 2C, 2E Figure S4C). AOX also does not revert the RP defects in *pink1* mutant animals (Figure 2F, 2G) nor does it alleviate the reduced red JC-1 labeling observed in mitochondria at boutons of *pink1* mutants (Figure 2H, 2I). Finally, also the reduced ATP levels, observed in *pink1* mutant animals, are not rescued by AOX (Figure 2D). Thus, AOX expression does not rescue *pink1* deficiency.

### Downregulation of a Complex I component phenocopies many *pink1* mutant phenotypes

**Figure 3:**
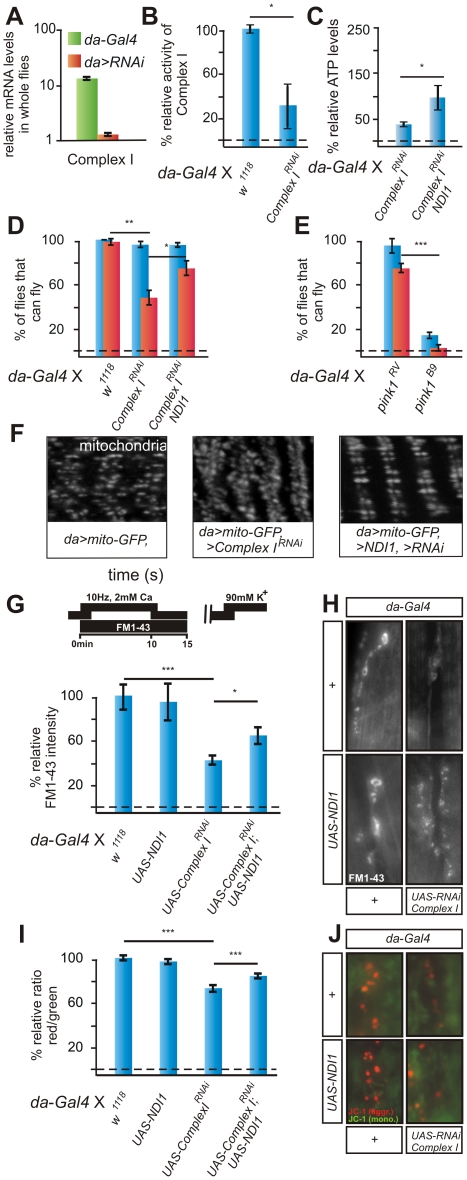
RNAi to a Complex I component (*CG12079*) phenocopies *pink1* mutants and is rescued by Ndi1p expression. (A) Quantitative RT-PCR using primers to *CG12079* in *w*; *da-Gal4*/*+* (green) and *w*; *UAS- CG12079^RNAi^*/*da-Gal4* (orange) larvae. Data normalized to housekeeping genes (see methods). (B) Complex I enzymatic activity measurement of whole third instar larval mitochondrial homogenates from *w* controls and Complex I deficient RNAi flies (*w*; *da-Gal4*/*UAS-CG12079^RNAi^*). Student's t-test: * *p*<0.05. Error bars SEM, n = 3 experiments with 50 larvae each. (C) Relative ATP levels of *w*; *UAS-CG12079^RNAi^*/*da-Gal4* normalized to control *w*; *da-Gal4/+* and of *w*; *UAS-NDI1*/+; *UAS-CG12079^RNAi^*/*da-Gal4* normalized to control flies *w*; *UAS-NDI1/+*; *da-Gal4/+*. Student's t-test: * *p*<0.05. Error bars SEM, n = 3 experiments with 5 larvae each. (D,E) Quantification of flight in *w*; *da-Gal4*/*+* controls, in Complex I RNAi expressing flies (*w*; *UAS-CG11455^RNAi^*; *da-Gal4*), in Complex I RNAi expressing flies that also express *NDI1* (*w*; *UAS-NDI1*, *UAS-CG11455^RNAi^*; *da-Gal4*) (D), in *pink1^RV^*controls and in *pink1^B9^* mutants (E) under ‘standard testing conditions’ (blue bars) and under ‘stringent testing conditions’ (red bars). Student's t-test: * *p*<0.05; ** *p*<0.01; *** *p*<0.001. Error bars SEM, n = 6 experiments with 5 flies each. (F) GFP labeling of mitochondria from the larval body wall muscles of controls (*w*; *da-Gal4, UAS-mito:GFP*/*+*), Complex I RNAi expressing flies (*w*; *da-Gal4, UAS-mito:GFP*/*UAS-CG12079^RNAi^*) and Complex I RNAi expressing flies that also express *NDI1* (*w*; *UAS-NDI1*; *da-Gal4, UAS-mito:GFP*/*UAS-CG12079^RNAi^*). (G,H) Quantification (G) and RP vesicle labeling (H) in controls (*w*; *da-Gal4*/+) in *w*; *da-Gal4*/*UAS-CG12079^RNAi^* and in *w*; *UAS-NDI1*; *da-Gal4*/*UAS-CG12073^RNAi^*. Student's t-test: * *p*<0.05; ns, not significant. Error bars SEM, n>8 synapses from 4 animals. (I,J) Quantification of red to green JC-1 fluorescence (I) and examples (J) at NMJ boutons in larvae of the genotypes indicated in (G–H). Student's t-test: *** *p*<0.001 Error bars SEM, n>16 synapses from 4 animals.

Our work suggests that several *pink1* mutant phenotypes stem from defects at the level of Complex I. To further test this hypothesis, we used RNAi mediated knock down of evolutionary conserved Complex I subunits (Figure 3A). As expected, down regulation of Complex I subunits results in significantly lower Complex I enzymatic activity (Figure 3B) and expression of *NDI1* in these flies results in an increased ATP concentration compared to RNAi of Complex I subunits in the absence of *NDI1* expression (Figure 3C). While expression of RNAi to some of the Complex I subunits results in developmental lethality, RNAi to other Complex I subunits yields adult flies, and we used one of those (*CG11455*; Gene ID: 33179) to assess flight upon Complex I knock down. Under ‘standard testing conditions’ (see methods) RNAi to this Complex I subunit does not result in a strong defect to fly (blue bar Figure 3D), however under more ‘stringent conditions’ (see methods) about half of the flies fail to fly, and this defect is rescued by expression of *NDI1* (red bars Figure 3D). Thus, while reduced Complex I activity results in a defect to fly, the flight deficit upon knock down of this Complex I subunit is milder than that observed in *pink1* mutants (Figure 3E).

Next, we determined mitochondrial morphology using Mito-GFP in third instar larval muscles upon knock down of a Complex I subunit. Similar to mitochondria in *pink1* mutants, mitochondria in muscles of animals that express RNAi to a Complex I subunit are swollen and clumped, but the defects we observe are in general less severe than those seen in *pink1^B9^* null mutants. Interestingly, expression of *NDI1* significantly alleviates these defects (Figure 3F). To assess mitochondrial function in animals that express RNAi to a Complex I subunit with or without *NDI1*, we assessed the mobilization of RP vesicles and we quantified red JC-1 labeling intensity within neuromuscular mitochondria. As indicated in Figure 3G–3J, reduced Complex I activity results in reduced RP vesicle mobilization and less red JC-1 labeling in boutonic mitochondria, and expression of *NDI1* can significantly rescue these defects as well. Thus, similar to *pink1* mutants, loss-of-function of a component of Complex I results in reduced ATP and functional defects in synaptic mitochondria, and these defects are alleviated by NDI1p. Furthermore, we also find that reduced Complex I activity leads to morphological defects in muscular mitochondria and a defect to fly in adult flies, but these defects are in general milder than those observed in *pink1* null mutants.

### Expression of NDI1 does not rescue *parkin* mutant phenotypes

**Figure 4:**
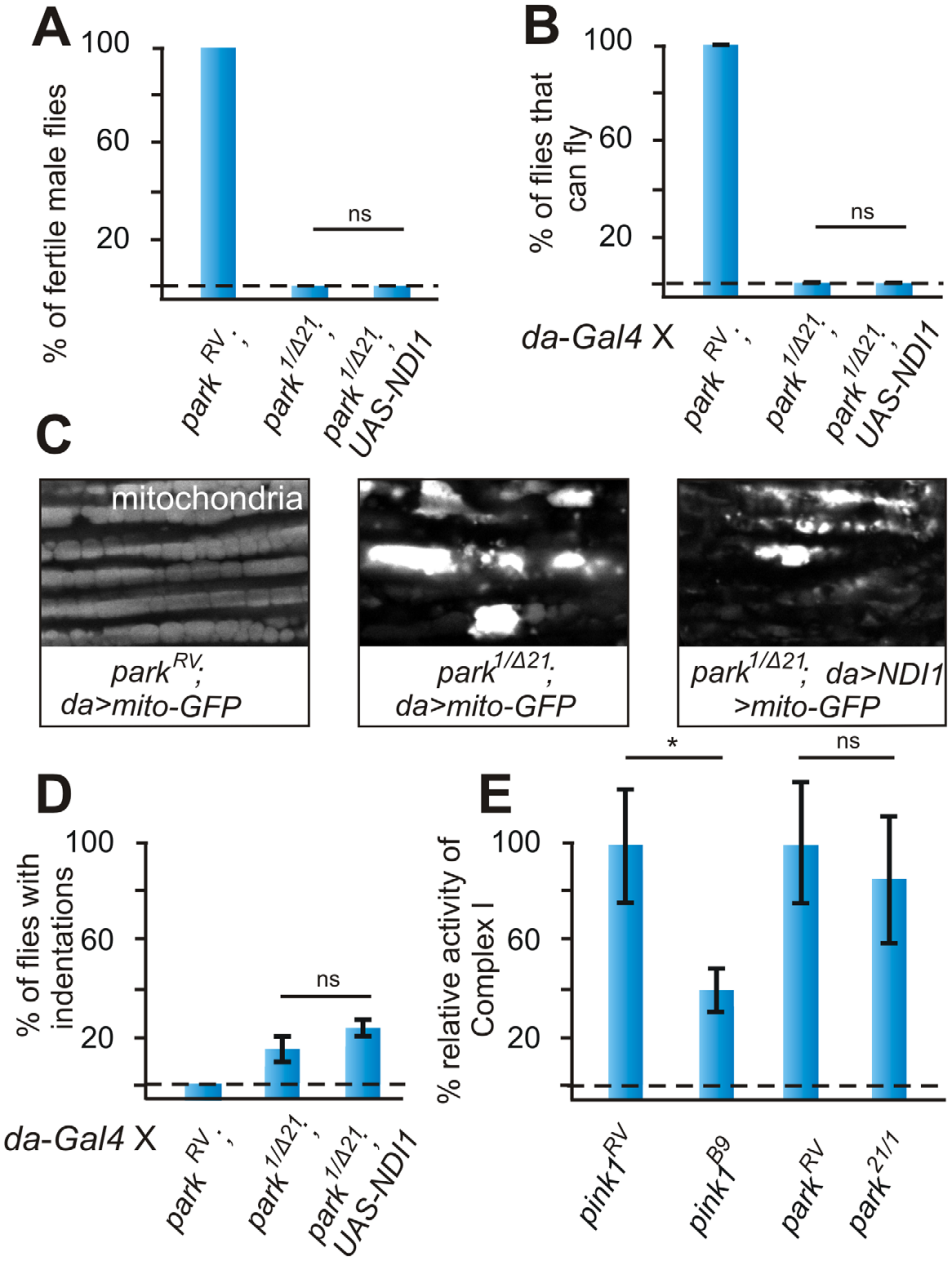
Ndi1p does not rescue *parkin* mutants. (A) Quantification of fertility of *w*; *parkin^RV^* control male flies of *w*; *park^1/Δ21^* and of *w*; *UAS-NDI1*; *park^1/Δ21^* male flies. “RV”: wild type *parkin* (precise *P* element excision); “1/Δ21”; mutant *park^1/Δ21^* heteroallelic combination (n = 30 animals). (B) Quantification of flight in control (*w*; *da-Gal4*, *park^RV^*) in *park^1/Δ21^* mutant (*w*; *da-Gal4*, *park^Δ21^*/*park^1^*) and in *park^1/Δ21^* mutant flies that express Ndi1p (*w*; *UAS-NDI1*/*+*; *da-Gal4*, *park^Δ21^*/*park^1^*). Student's t-test: * *p*<0.05. Error bars SEM, n = 10 experiments with 5 flies each. (C) GFP labeling of mitochondria from the adult indirect flight muscles of the following genotypes *UAS-mito:GFP*/*+*; *da-Gal4*, *park^RV^* and *UAS-mito:GFP*/*+*; *da-Gal4*, *park^Δ21^*/*park^1^* and *UAS-mito:GFP/UAS-NDI1*; *da-Gal4*, *park^Δ21^*/*park^1^*. (D) Quantification of thorax indentations of genotypes indicated in (A). Student's t-test: * *p*<0.03. Error bars SEM, n = 10 experiments with 5 flies each. (E) Complex I enzymatic activity measurement of whole fly-mitochondrial homogenates from *pink1^RV^* controls and *pink1^B9^* mutants compared to *park^RV^* controls and *park^1/Δ21^* mutants. Student's t-test: * *p*<0.05; ns, not significant. Error bars SEM, n>6 experiments with 50 flies each.

Pink1 has been suggested to act upstream of Parkin to regulate, in a linear pathway, mitophagy [8]–[10], [39], [40]. We therefore expressed Ndi1p in *parkin* mutant flies but we did not observe a rescue of male fertility, flight defects or muscular degeneration (Figure 4A, 4B, 4D). In line with these observations, mitochondrial morphological alterations caused by Parkin deficiency are also not rescued by expression of Ndi1p (Figure 4C). Thus, *parkin* mutants cannot be rescued by expression of Ndi1p.

To determine if *parkin* mutants also display reduced enzymatic activity of Complex I we isolated mitochondria from *parkin* null mutant flies and from controls and measured Complex I enzymatic activity. In contrast to *pink1* mutants, the isolated enzymatic activity of Complex I in *parkin* mutant mitochondria is similar to controls (Figure 4E). These data are in further support of a model where Complex I defects in *pink1* mutants occur upstream from the defects caused by loss of Parkin function.

### Modulation of mitochondrial morphology does not rescue Complex I defects in *pink1* mutants

**Figure 5:**
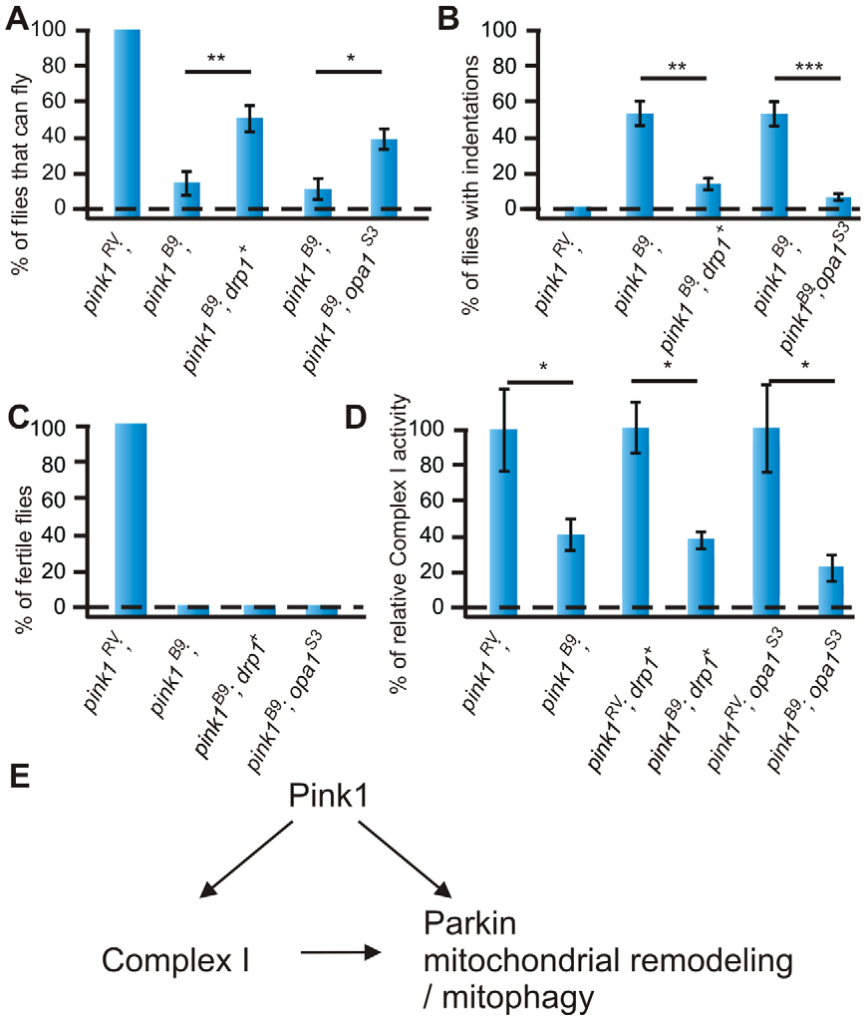
Increased mitochondrial fission does not rescue reduced Complex I enzymatic activity of *pink1* mutants. (A) Quantification of flight in control (*w pink1 ^RV^*) in *pink1^B9^* (*w pink1^B9^*) and in *pink1^B9^* mutant flies that either have a genomic rescue construct of *drp1* (*drp1^+^*) or have one mutant allele of *opa1* (*opa1^S3^*) (*w pink1^B9^*; *drp1^+^*/+ and *w pink1^B9^*; *opa1^S3^*/+). Student's t-test: ** *p*<0.01; * *p*<0.05. Error bars SEM, n = 10 experiments with 5 flies each. (B) Quantification of thorax indentations of genotypes indicated in (A). Student's t-test: ** *p*<0.01; *** *p*<0.001. Error bars SEM, n = 10 experiments with 5 flies each. (C) Quantification of fertility of *w pink1^RV^* control male flies, *w pink1^B9^* mutants and of *w pink1^B9^*; *drp1^+^*/+ and of *w pink1^B9^*; *opa1^S3^*/+ resp. male flies (n = 30 animals). (D) Complex I enzymatic activity measurement of whole fly mitochondrial homogenates from *w pink1^RV^* controls and *w pink1^B9^* mutants compared to *w pink1^RV^*; *drp1^+^*/+ controls, *w pink1^B9^*; *drp1^+^*/+ mutants and *w pink1^RV^*; *opa1^S3^*/+ controls, *w pink1^B9^*; *opa1^S3^*/+ mutants. Student's t-test: * *p*<0.05. Error bars SEM, n>3 experiments with 50 flies each. The data for *w pink1^RV^* and *w pink1^B9^* is identical to that shown in Figure 4E. (E) Model of Pink1 and Parkin function in the regulation of mitochondrial activity. Our work indicates that Pink1 has an important function at the level of Complex I, and these defects in part result in mitochondrial remodeling defects seen in the mutants.

Previous reports indicate that genetic manipulation of the mitochondrial remodeling machinery using over expression of *drp1* or loss-of-function of *opa1* alleviates mitochondrial morphological defects, muscle degeneration and flight deficits both in *pink1* and in *parkin* mutant flies [17], [19] and we confirm these results (Figure 5A, 5B, data not shown). In contrast however, sterility of *pink1* mutant males is not rescued by increased *drp1* or decreased *opa1* (Figure 5C), suggesting that manipulation of mitochondrial remodeling cannot rescue all *pink1*-related phenotypes. As mitochondrial morphological phenotypes may result from alterations in numerous biochemical pathways [26], we assessed directly whether *drp1* or *opa1* affect the enzymatic activity of Complex I in *pink1* mutant flies. However, as shown in Figure 5D, the enzymatic activity of Complex I is still reduced to a level similar to that observed in *pink1* mutants. These data thus indicate that the enzymatic deficiency at the level of Complex I in *pink1* mutants precedes mitochondrial morphological deficits, or that they in part occur independently.

## Discussion

In this work we present compelling evidence that the mitochondrial kinase Pink1 is critically required to maintain efficient Complex I enzymatic activity in mitochondria and that this function precedes mitochondrial remodeling or mitophagy (Figure 5E). While Pink1 likely acts via multiple (phospho-) targets [41], [42], our data suggests a pathway in which many of the deficiencies in *pink1* can be traced back to mitochondrial dysfunction [8]–[10], [15].

Our experiments indicate that Pink1 acts at, or in parallel to, Complex I, in line with the reduced enzymatic activity of this complex in *pink1* mutant mouse cells and flies [15], [23]. Expression of Ndi1p alleviates many *pink1*-associated phenotypes, suggesting that more efficient electron transport between NADH and ubiquinone is mediated by Ndi1p (bypassing the endogenous Complex I deficiency) [30] in *pink1* mutants that boosts formation of a proton gradient by Complex III and IV. Although AOX expression also improves ETC efficiency [32], it does not rescue *pink1*-associated phenotypes, in contrast to AOX rescuing *DJ-1β* (CG1349 Gene ID: 43652) and *cyclope* associated phenotypes [32]. The lack of rescue is likely not due to the fact that AOX is insufficiently activated as a result of low reduced ubiquinone concentrations [43] as expression of AOX in *pink1* mutants results in premature death of *pink1* animals such that only few *pink1* mutants that express AOX emerge as adults (data not shown). We surmise that the lower Complex I activity in *pink1* mutants, which results in reduced proton transfer across the inner mitochondrial membrane [15] is further propagated by the presence of AOX, that transfers electrons to oxygen without pumping protons [29]. These data thus argue against general mitochondrial dysfunction in *pink1* and a universal failing of the ETC [23] but reveals an important role for Pink1 upstream or in parallel to Complex I enzymatic activity [15].

Previous work indicates that loss of *pink1* in some cell types results in mitochondrial fragmentation, a process preceding mitophagy [44]. Several lines of evidence now indicate that some of the mitochondrial morphological defects occur downstream of functional deficits in *pink1* mutants. First, we show that expression of Ndi1p in *pink1* mutants alleviates part of the mitochondrial morphological defects. Second, RNAi-mediated knock down of an evolutionary conserved Complex I component results in mitochondrial dysfunction but also mitochondrial swelling and clumping, indicating that functional mitochondrial defects can lead to morphological defects [24], [26], [45]. Third, mitochondrial functional defects in *pink1* mutant flies and mice have been widely observed in neuronal populations where mitochondrial morphological defects are not (yet) prevalent [15], [23]. Fourth, while facilitating mitochondrial fission in *pink1* mutants alleviates mitochondrial morphological defects, a deficiency at the level of the enzymatic activity of Complex I persists. Previous results in *pink1* knock out cells had also indicated that mitochondrial swelling defects in *pink1* mutant cells can be rescued by modulating the levels of the mitochondrial fission machinery [46], but also this manipulation failed to rescue the defect in mitochondrial membrane potential caused by loss of Pink1 [47]. Thus, our data indicate that the upstream molecular dysfunction in *pink1* mutants on Complex I is a major culprit in the development of the *pink1* mutant phenotypes (Figure 5E).

Our rescue experiments indicate that expression of *NDI1* can significantly rescue numerous *pink1* associated phenotypes, including male sterility, vesicle trafficking and mitochondrial membrane potential. Likewise, *NDI1* also alleviates mitochondrial morphological defects in *pink1* mutant muscles, but rescue of this morphological defect is only partial. Similarly, mitochondrial morphological defects observed in animals that express RNAi to a Complex I subunit and flight defects in such animals are in general less severe than those seen in *pink1* null mutants. These results are consistent with Pink1 also acting in parallel to its role at the level of Complex1; however, we cannot exclude the possibility that the partial rescue of morphological defects in *pink1* mutants upon expression of *NDI1* originates from an incomplete reconstitution of ETC activity under these conditions, and that the *pink1* mutant conditions at the level of Complex I may not be exactly recapitulated by knock down of the Complex I components. Furthermore, given that Complex I dysfunction results in mitochondrial morphological defects and mild flight defects that are rescued by *NDI1* expression, and the observation that the enzymatic defects at the level of Complex I in *pink1* mutants are not rescued upon expression of Drp1 or loss of *opa1* function, the data are consistent with the pink1-associated Complex I defects to act at least in part upstream of remodeling and suggest an important and central role for Complex I in Pink1 induced mitochondrial pathology.

Our work expands on previous genetic and cell biological studies, and indicates that Pink1 can act at a different level, upstream of Parkin, to control Complex I enzymatic activity (Figure 5E). Indeed, unlike *pink1* mutants, loss of *parkin* in flies does not cause significantly reduced enzymatic activity of Complex I seen in *pink1* mutants, and in addition, *parkin* loss of function is not rescued by expression of Ndi1p. Our data indicate that Complex I deficiency in *pink1* mutants is specific and that this defect is not a result of abnormal mitochondrial remodeling or mitophagy. This model is consistent with a role of Pink1 in controlling mitochondrial health and also does not exclude a downstream or parallel role where Pink1 can be triggered to recruit Parkin, facilitating mitophagy (Figure 5E).

## Materials and Methods

### *Drosophila* stocks and maintenance

*w; UAS-mito:GFP*, *w; da-Gal4* and *w; P{lacW}opa1-like^3475^/CyO (opa1^S3^)* were obtained from Bloomington stock center (Indiana, USA); *w pink1^B9^* and *w pink1^RV^*, *parkin^1^* and *parkin^RV^* [48] were gifts from Jongkyeong Chung (Korea, Advanced Institute of Science and Technology) [9] and *parkin^Δ21^* mutant flies were a gift from Graeme Mardon (Baylor College of Medicine) [11]. *drp1^+^* genomic rescue constructs were provided by Hugo Bellen (Baylor College of Medicine) [16] and *w^1118^; UAS-AOX^F6^* were a gift from Howard T. Jacobs [32] (Finland, Institute of Medical Technology, Tampere University Hospital). *w^1118^; UAS-CG12079^RNAi^* (*w^1118^; P{GD5910}v13856*), *w^1118^; UAS-CG11455^RNAi^* (*w^1118^; P{GD4800}v12838*) and *cyclope* (*CG14028*) RNAi (*w^1118^; P{GD908}v13403*) were from the Vienna *Drosophila* RNAi Center (VDRC) [49]. Flies were raised on standard cornmeal and molasses medium.

### Generation of *UAS-NDI1* transgenic flies

The coding region of *Saccaromyces cerevisiae NDI1* was amplified from yeast genomic DNA (Patrick Van Dijck, VIB Leuven), with the following primers; CGG AAT TCC AAA ATG CTA TCG AAG and GGC GGC CGC CTA TAA TCC TTT A using 2 X BIO-X-ACT Short Mix (BIOLINE), cloned in the EcoR1 and Not1 of *pUAST-Attb* (43) and sequenced. Transgenic flies were created at GenetiVision Inc. (Houston, USA) using PhiC31 mediated transgenesis in the VK1 docking site (2R, 59D3) [50].

### Quantitative RT–PCR

Testis samples from *w; daGal4*/+ and *w; UAS-NDI1* and *w; UAS-NDI1/+; da-Gal4/+* and *w; UAS-AOX^F6^* and *w; UAS-AOX^F6^/+; da-Gal4/+* adult flies were micro-dissected in ice-cold PBS followed by snap-freezing on dry ice. Third instar larvae from *w*; *daGal4*/+ and *w*; *UAS-CG12079^RNAi^*/*da-Gal4* were also snap-frozen on dry ice. Q-RT-PCR was performed using standard procedures, as outlined in Text S1, and relative RNA levels were calculated according to the ΔΔCt method. Primers are listed in Table S1.

### Sterility test

Single males (30) of the genotypes indicated in the figure legends were crossed to 1–3 day old *w^1118^* virgins and a score of 1 was assigned when offspring was detected.

### Flight assays

For flight assays, batches of 5 freshly eclosed male flies grown at 18°C of the genotypes indicated in the figure legends were put at room temperature for 2 days and then transferred to an empty vial (5 cm D, 10 cm H). For ‘standard testing conditions’, flies were allowed to climb above a marked line at 9 cm height, the vial was gently tapped and visually scored for flying flies over the next minute. For more ‘stringent testing conditions’ flies were allowed to climb above a marked line at 9 cm height, and flying flies were scored immediately following tapping of the vial. Flies at the bottom were removed and the remaining flies were retested. Flies that fly twice were assigned a score of 1, the others a score of 0. Student's t test was used to assess the statistical differences.

### ATP measurements

Five third instar larvae or five thoraces from 2–3 days-old flies were dissected and homogenized in 50 µl of 6 M guanidine-HCl 100 mM Tris and 4 mM, EDTA, pH 7.8. These homogenates were snap-frozen in liquid nitrogen and then boiled for 3 min. Samples were then centrifuged and the supernatant was diluted (1/50) in extraction buffer, mixed with luminescent solution (ATP Determination Kit, Invitrogen) and luminescence was measured on an EnVision Multilabel Reader (Perkin Elmer). Luminiscence was normalized to protein amount (mg) (Bradford) and compared to ATP standards. n = 3. Student's t test was used to assess the statistical differences.

### Microscopy

Thoraces of adult flies were viewed under an Olympus SZX12 microscope equipped with a DF PLAPO 1X PF lens and the pictures were captured with an Olympus U-CMAD3 camera.

Mitochondrial morphology in adult flight muscles of flies grown at 18°C and reared at room temperature for 2 days or in wandering third instar larvae grown at 25°C was assessed by visualizing mitochondrial tagged GFP (mito-GFP), excited using 488 nm laser light and imaged on a Zeiss LSM 510 META confocal microscope using a 63×oil NA 1.4 lens (for adult muscles) or a 40×oil NA 1.3 lens (for larvae) using a 500–530 band pass emission filter.

RP vesicles of larvae were labeled by electrically stimulating motor neurons of third instar larval fillets in HL-3 with 2 mM Ca^2+^ for 10 min at 10 Hz and then leaving the preparation to rest for 5 min in the presence of the dye following stimulation. This protocol labels the entire vesicle pool; the exo-endo cycling pool (ECP) and the RP. To unload the FM 1–43 from the ECP, leaving RP labeling intact, preparations were subsequently incubated for 5 min in HL-3 with 90 mM KCl and 2 mM Ca^2+^ (in the absence of FM 1–43) [16]. Following washing in Ca^2+^ free HL-3, NMJs were imaged on a Nikon FN-1 microscope with a DS-2MBWc digital camera, 40×W NA 0.8 objective and quantification of labeling intensity was performed using NIS-Elements AR 3.10.

JC-1 (Molecular Probes) labeling was performed on wandering third instar larvae of the genotypes indicated in the figure legends as described previously [15]. Red and green fluorescence was captured on a Nikon FN-1 microscope with a Hamamatsu ORCA-*R* ^2^, 40W NA 0.8 objective. Quantification of red and green labeling intensity was performed using NIS-Elements AR 3.10.

### Enzymatic Complex I activity measurements

Complex I activity measurements were performed as described [15]. Data represent at least 3 independent experiments where mitochondrial preparations from 50 animals were prepared for each independent experiment. The Complex I enzymatic activity was normalized to Citrate Synthase enzymatic activity.

## Supporting Information

Figure S1 Expression of *NDI1* is benign and rescues Complex I defects. (A) Quantitative RT-PCR using primers to *CG3446*, a component of ETC Complex I and to *NDI1*, in whole flies using tissue of the following genotypes *w*; *da-Gal4* (green), *w*; *UAS-NDI1* (orange) and *w*; *UAS-NDI1*; *da-Gal4* (blue). Data normalized to housekeeping genes (Text S1). (B) Complex I enzymatic activity measurements in mitochondrial homogenates of control (*w pink1^RV^*; *da-Gal4*), *w*; *UAS-NDI1*; *da-Gal4*, *w pink1^B9^*; *da-Gal4* and *w pink1^B9^*; *UAS-NDI1*; *da-Gal4* in the absence of Rotenone (green) and presence of Rotenone (orange). Student's t-test: * *p*<0.05; ** *p*<0.01; ns = non-significant. Data represent the average +/− SEM of n = 3 experiments with 50 flies each. (C) Quantification of flight in *w*; *UAS-NDI1*; *da-Gal4* flies and *w*; *da-Gal4/+* controls. Data represent the average +/− SEM of n = 10, (5 flies per independent test assayed). ns = non-significant. (D) Quantification of thorax indentations in *NDI1* expressing flies: *w*; *UAS-NDI1*; *da-Gal4* and *da-Gal4/+* controls. Data represent the average +/− SEM of n = 6 (5 flies per independent test assayed). ns = non-significant.
(TIF)

Figure S2 Expression of *NDI1* rescues sterility and indentation phenotypes of *pink1* mutant flies. (A) Images of hemizygous male *w pink1^RV^*, *w pink1^B9^* and *w pink1^B9^*; *UAS-NDI1* flies and of homozygous female *w pink1^RV^* and *w pink1^B9^*; *UAS-NDI1* flies. na = not applicable. (B) PCR on genomic DNA from *w pink1^RV^* males (lane 1), *w pink1^B9^*/*+* heterozygous females (lane 2), *w pink1^B9^* hemizygous males (lane 3) and *w pink1^B9^*; *UAS-NDI1* homozygous females (lane 4) using primers to amplify the *pink1* locus, the *NDI1* transgene and *male fertility factor kl3* on the Y chromosome (Text S1). Primers used are listed in Table S1. (C) Quantification of thorax indentations in control (*w pink1^RV^*; *da-Gal4*/*+*) in *pink1^B9^* mutant (*w pink1^B9^*; *da-Gal4*/*+*) and in *pink1^B9^* mutant flies that express Ndi1p (*w pink1^B9^*; *UAS-NDI1*/*+*; *da-Gal4*/*+*). Student's t-test: * *p*<0.05. Data represent the average +/− SEM of n = 10 experiments with 5 flies each.
(TIF)

Figure S3 Expression of *NDI1* rescues *pink1* mutant defects in neurotransmitter release. (A) Quantification of the amplitude of excitatory junctional potentials measured in 2 mM external calcium in *pink1^RV^* controls (*w pink1^RV^*; *da-Gal4/+*), in larvae expressing *NDI1* (*w*; *UAS-NDI1/+*; *da-Gal4/+*), in *pink1^B9^* mutants (*w pink1^B9^*; *da-Gal4/+*) and in *pink1^B9^* mutants expressing *NDI1* (*w pink1^B9^*; UAS*-NDI1/+*; *da-Gal4/+*). Student's t-test: ns = non-significant. Data represent the average +/− SEM of n = 4 animals (8 NMJs). (B) Relative EJP amplitudes measured in 2 mM Ca^2+^ during 10 min of 10 Hz stimulation in controls, *pink1^B9^* mutants, *pink1^B9^* mutants expressing *NDI1* and controls expressing *NDI1* (genotypes, see A). EJP amplitudes were binned per 30 s and normalized to the average amplitude of the first 10 EJPs. Data represent the average +/− SEM of n = 4 animals (8 NMJs).
(TIF)

Figure S4 Expression of *AOX* is benign. (A) Quantification of flight in *w*; *UAS-AOX*/*+*; *da-Gal4*/*+* and in *w*; *da-Gal4/+* controls. Data represent the average +/− SEM of n = 6 (5 flies per independent test). Student's t-test: ns = non-significant. (B) Quantification of indentations in *w*; *UAS-AOX*/*+*; *da-Gal4*/*+* and in *w*; *da-Gal4/+* controls. Data represent the average +/− SEM of n = 6 (5 flies per independent test). Student's t-test: ns = non-significant. (C) Quantification of thorax indentations in control (*w pink1^RV^*; *da-Gal4/+*) in *pink1^B9^* mutant (*w pink1^B9^*; *da-Gal4/+*) and in *pink1^B9^* mutant flies that express *AOX* (*w pink1^B9^*; *UAS-AOX/+*; *da-Gal4/+*). Student's t-test: ns = non-significant. Data represent the average +/− SEM of n = 6 experiments with 5 flies each.
(TIF)

Table S1 Primers used for Q-RT-PCR and PCR from genomic DNA. List of primers used for Q-RT-PCR and of primers used for PCR of genomic DNA.
(DOC)

Text S1 Supplemental methods.
(DOC)
