# Supplementary figures and images for: The Yeast Complex I Equivalent NADH Dehydrogenase Rescues pink1 Mutants
Source: PLoS Genet. 2012 Jan 5;8(1):e1002456. doi: 10.1371/journal.pgen.1002456 (PMC3252300; doi:10.1371/journal.pgen.1002456)

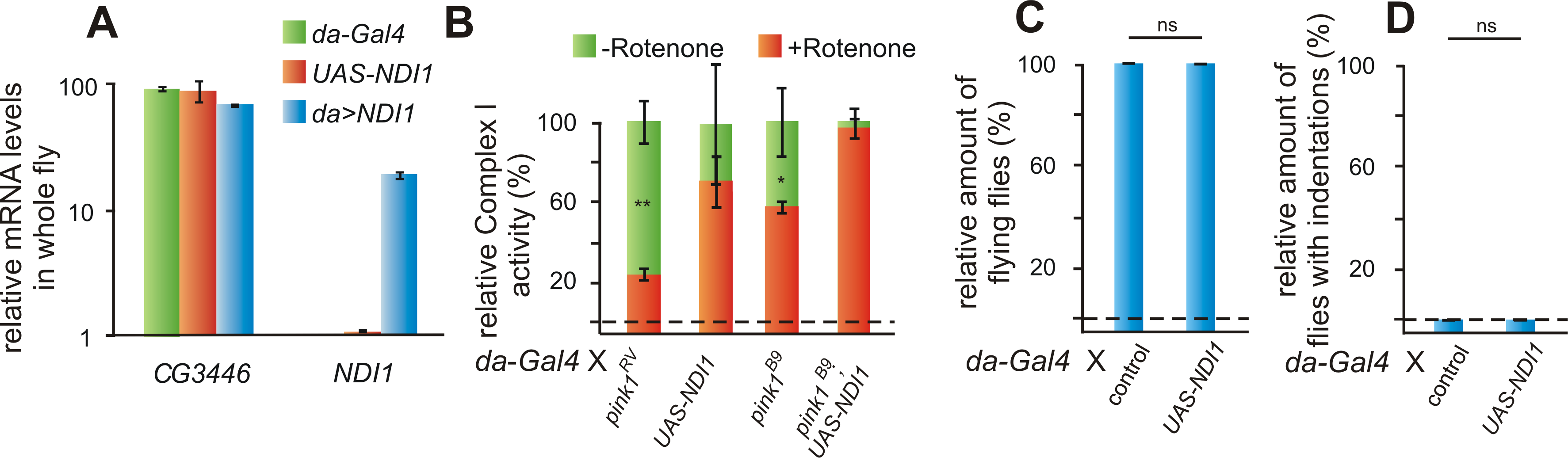

Supplement: Figure S1 — Expression of NDI1 is benign and rescues Complex I defects. (A) Quantitative RT-PCR using primers to CG3446, a component of ETC Complex I and to NDI1, in whole flies using tissue of the following genotypes w; da-Gal4 (green), w; UAS-NDI1 (orange) and w; UAS-NDI1; da-Gal4 (blue). Data normalized to housekeeping genes (Text S1). (B) Complex I enzymatic activity measurements in mitochondrial homogenates of control (w pink1RV; da-Gal4), w; UAS-NDI1; da-Gal4, w pink1B9; da-Gal4 and w pink1B9; UAS-NDI1; da-Gal4 in the absence of Rotenone (green) and presence of Rotenone (orange). Student's t-test: * p<0.05; ** p<0.01; ns = non-significant. Data represent the average +/− SEM of n = 3 experiments with 50 flies each. (C) Quantification of flight in w; UAS-NDI1; da-Gal4 flies and w; da-Gal4/+ controls. Data represent the average +/− SEM of n = 10, (5 flies per independent test assayed). ns = non-significant. (D) Quantification of thorax indentations in NDI1 expressing flies: w; UAS-NDI1; da-Gal4 and da-Gal4/+ controls. Data represent the average +/− SEM of n = 6 (5 flies per independent test assayed). ns = non-significant. (TIF) [file pgen.1002456.s001.tif]

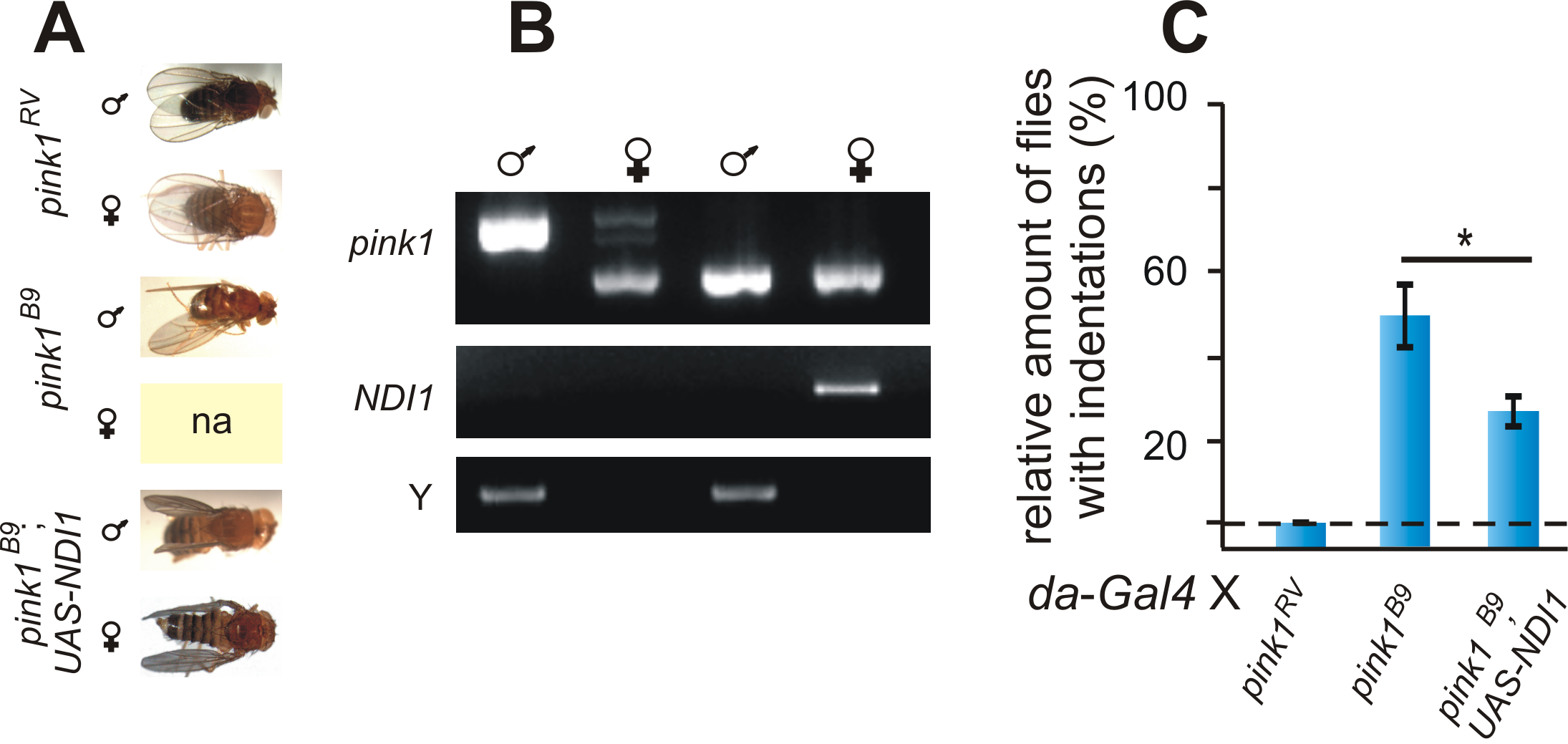

Supplement: Figure S2 — Expression of NDI1 rescues sterility and indentation phenotypes of pink1 mutant flies. (A) Images of hemizygous male w pink1RV, w pink1B9 and w pink1B9; UAS-NDI1 flies and of homozygous female w pink1RV and w pink1B9; UAS-NDI1 flies. na = not applicable. (B) PCR on genomic DNA from w pink1RV males (lane 1), w pink1B9/+ heterozygous females (lane 2), w pink1B9 hemizygous males (lane 3) and w pink1B9; UAS-NDI1 homozygous females (lane 4) using primers to amplify the pink1 locus, the NDI1 transgene and male fertility factor kl3 on the Y chromosome (Text S1). Primers used are listed in Table S1. (C) Quantification of thorax indentations in control (w pink1RV; da-Gal4/+) in pink1B9 mutant (w pink1B9; da-Gal4/+) and in pink1B9 mutant flies that express Ndi1p (w pink1B9; UAS-NDI1/+; da-Gal4/+). Student's t-test: * p<0.05. Data represent the average +/− SEM of n = 10 experiments with 5 flies each. (TIF) [file pgen.1002456.s002.tif]

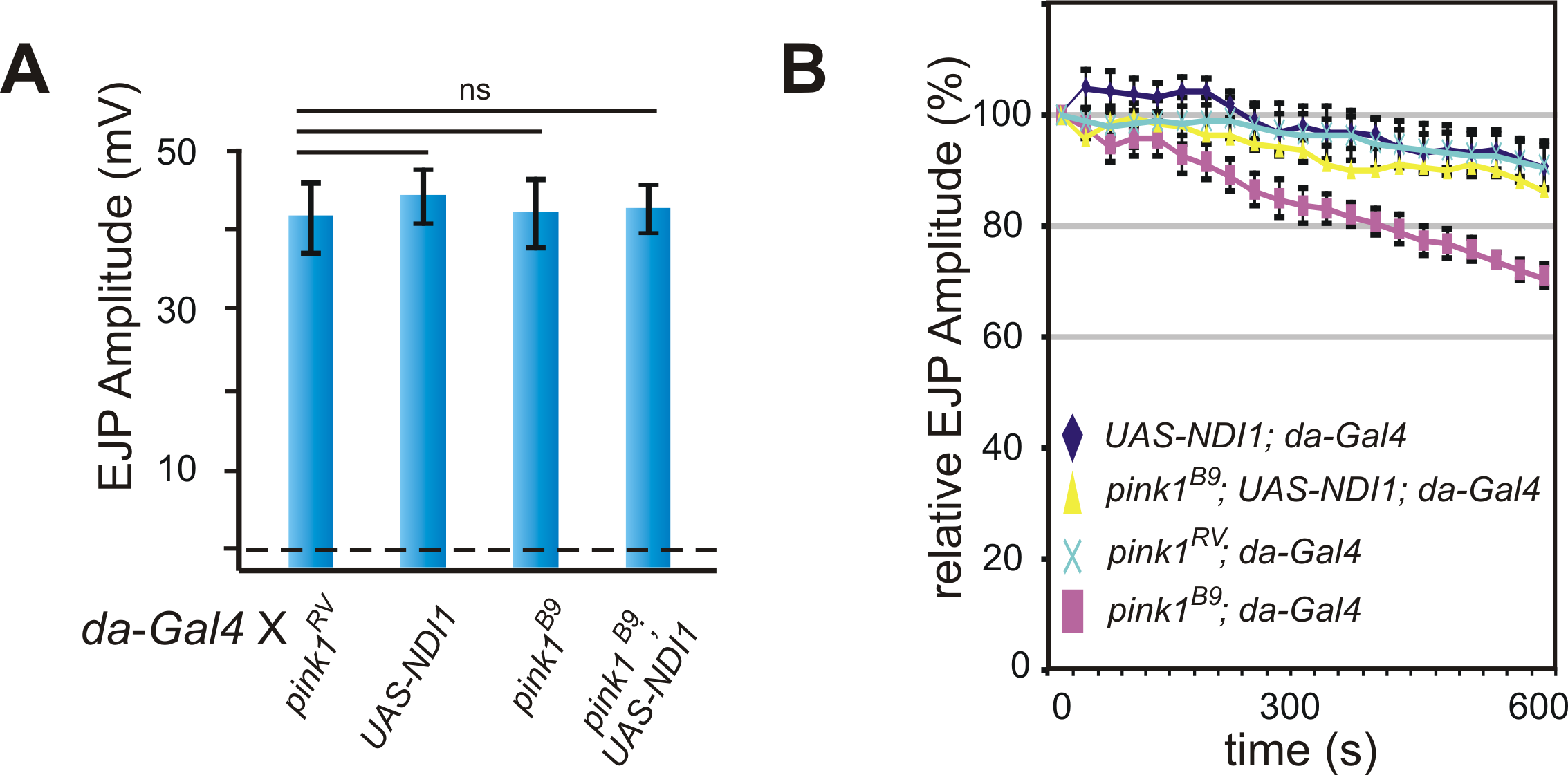

Supplement: Figure S3 — Expression of NDI1 rescues pink1 mutant defects in neurotransmitter release. (A) Quantification of the amplitude of excitatory junctional potentials measured in 2 mM external calcium in pink1RV controls (w pink1RV; da-Gal4/+), in larvae expressing NDI1 (w; UAS-NDI1/+; da-Gal4/+), in pink1B9 mutants (w pink1B9; da-Gal4/+) and in pink1B9 mutants expressing NDI1 (w pink1B9; UAS-NDI1/+; da-Gal4/+). Student's t-test: ns = non-significant. Data represent the average +/− SEM of n = 4 animals (8 NMJs). (B) Relative EJP amplitudes measured in 2 mM Ca2+ during 10 min of 10 Hz stimulation in controls, pink1B9 mutants, pink1B9 mutants expressing NDI1 and controls expressing NDI1 (genotypes, see A). EJP amplitudes were binned per 30 s and normalized to the average amplitude of the first 10 EJPs. Data represent the average +/− SEM of n = 4 animals (8 NMJs). (TIF) [file pgen.1002456.s003.tif]

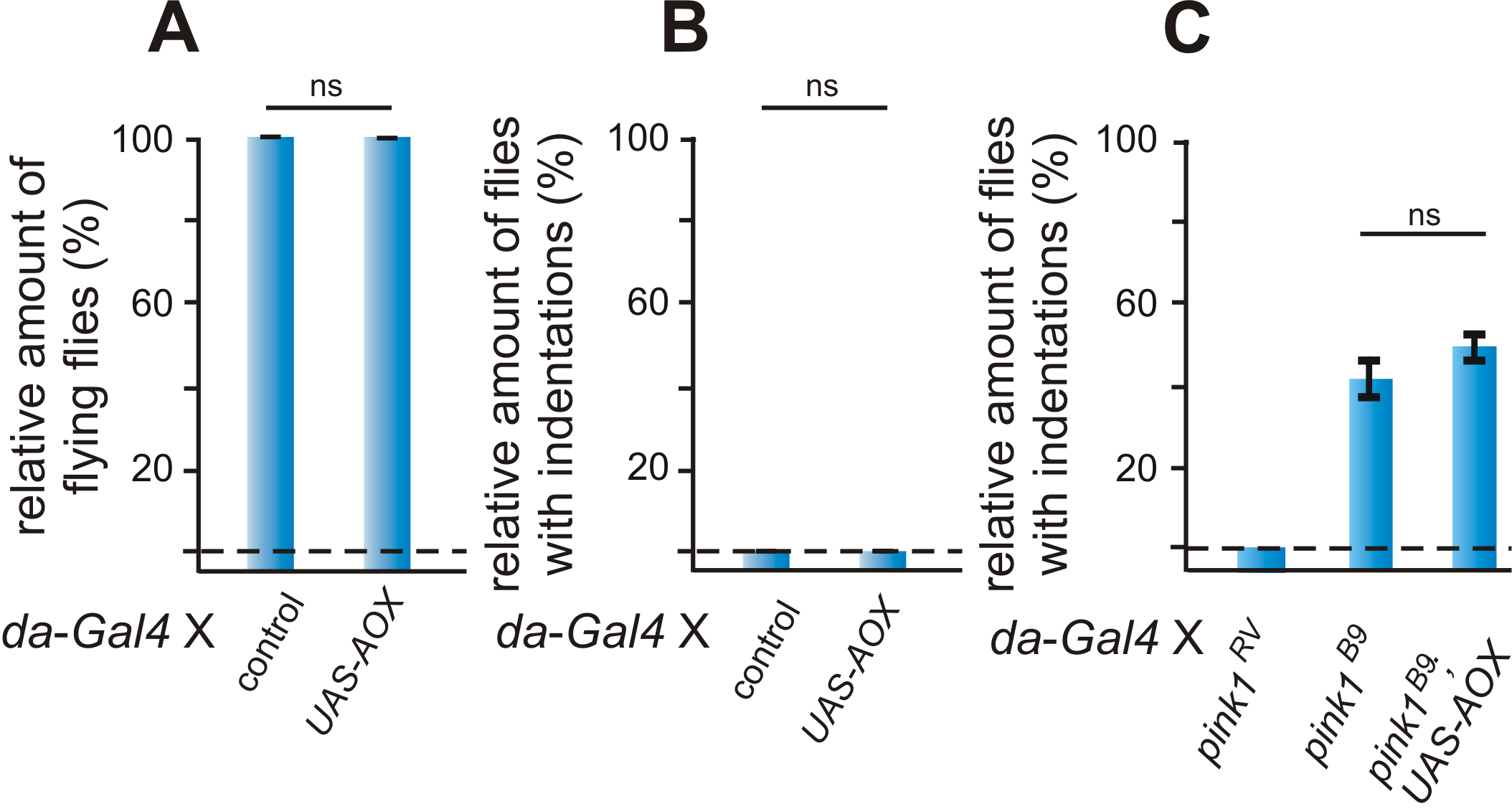

Supplement: Figure S4 — Expression of AOX is benign. (A) Quantification of flight in w; UAS-AOX/+; da-Gal4/+ and in w; da-Gal4/+ controls. Data represent the average +/− SEM of n = 6 (5 flies per independent test). Student's t-test: ns = non-significant. (B) Quantification of indentations in w; UAS-AOX/+; da-Gal4/+ and in w; da-Gal4/+ controls. Data represent the average +/− SEM of n = 6 (5 flies per independent test). Student's t-test: ns = non-significant. (C) Quantification of thorax indentations in control (w pink1RV; da-Gal4/+) in pink1B9 mutant (w pink1B9; da-Gal4/+) and in pink1B9 mutant flies that express AOX (w pink1B9; UAS-AOX/+; da-Gal4/+). Student's t-test: ns = non-significant. Data represent the average +/− SEM of n = 6 experiments with 5 flies each. (TIF) [file pgen.1002456.s004.tif]
