# Supplementary material for: The Yeast Complex I Equivalent NADH Dehydrogenase Rescues pink1 Mutants
Source: PLoS Genet. 2012 Jan 5;8(1):e1002456. doi: 10.1371/journal.pgen.1002456 (PMC3252300; doi:10.1371/journal.pgen.1002456)
Supplement: Table S1 — Primers used for Q-RT-PCR and PCR from genomic DNA. List of primers used for Q-RT-PCR and of primers used for PCR of genomic DNA. (DOC) [file pgen.1002456.s005.doc]

**Supplemental Table**

Supplemental Table 1. Primers used for Q-RT-PCR and PCR from genomic DNA

| Primers used for Q-RT-PCR | | |
| --- | --- | --- |
| Target gene | Forward sequence | Reverse sequence |
| *RPL32* | GGTATGTGCGTGATTTTGGG | TCCCATCACAAACAGAAGCC |
| *Act79B* | CCGCACCAAACTAACCAAAC | TCCTTCTGACCCATACCCA |
| *NDI1* | GAAGCCACCTCTATCAATCCC | AATGACCGTAATCAGTGACCC |
| *CG3446* | GCTACTTCACAGGCTTCACC | TGGGAAAGATGACATTCTGGG |
| *βTUB85D* | GTCCGAGGGATGCGATTG | CTCAGGGTGGCATTGTAGG |
| *GstS1* | CTGGAGAGAGTGAGAGTAGAGAG | AGTTCATGTAGTCGGTGATGC |
| *AOX* | GTTGGCTATCTGGAAGAGGAAG | ATTGCCAGGATAACGTCTCG |
| *CG12079* | CACTCGGCCACATAGCG | TTAATCAGGAATCTGGGTGCC |
| Primers used for PCR from genomic DNA | | |
| *pink1* | GATCCAGCTGACCAGCGATTA | GATACAGCGACATATTGCGG |
| *kl3* (Y chr) | GTAACGATACACGTACATCAG | GTCGCATACTTTGATAATCGC |
