# Supplementary material for: The Yeast Complex I Equivalent NADH Dehydrogenase Rescues pink1 Mutants
Source: PLoS Genet. 2012 Jan 5;8(1):e1002456. doi: 10.1371/journal.pgen.1002456 (PMC3252300; doi:10.1371/journal.pgen.1002456)
Supplement: Text S1 — Supplemental methods. (DOC) [file pgen.1002456.s006.doc]

**Supplemental Methods for Vilain et al.**

**RT-PCR**

RNA was isolated using the RNeasy Micro Kit (Qiagen). Reverse transcription was performed using SuperScript® III Reverse Transcriptase (Invitrogen) and a mixture of poly-dT primers and random hexamers. Quantitative RT-PCR was performed using 25 ng target cDNA, a specific primer pair (IDT, Haasrode, Belgium) (Supplemental Table 1), Platinum SYBR Green qPCR SuperMix-UDG with ROX (Invitrogen), and water on an ABI7000. All wells were analyzed in duplicate. The primers were designed with the online tool of IDT and in-silico validated using the tools of RTPrimerDataBase. *Act79B* (Gene ID: 40444) and *RPL32* (Gene ID: 43573) were used as reference genes for the whole fly samples, and *βTubulin85D* (Gene ID: 41124) and *GstS1* (Gene ID: 36927) for the testis samples.

**Genomic PCR**

To test the presence of the *pink1* mutation, the presence of *NDI1* or the Y chromosome, single flies were homogenized in squishing buffer (10 mM Tris-HCl pH 8, 1 mM EDTA 25 mM NaCl, 200 g/ml proteinase K), incubated at 55°C for 1h and heated at 95°C for 2 minutes to inactivate proteinase K. Genomic DNA was diluted to 10ng/µl. Primers used are listed in Supplemental Table 1; for detection of the Y chromosome we amplified part of *male fertility factor kl3* (Gene ID: 5740653).

**Electrophysiology**

Excitatory junctional potentials were recorded in modified HL-3 with 2 mM CaCl_2_ as described [1,2]. Recordings were taken from muscle 6 in segment A2 or A3 with a sharp intracellular electrode and input resistances were >5MΩ.

**Thoracic indentations**

Freshly eclosed male flies grown at 18°C were pooled in batches of five flies and analyzed under a stereomicroscope for the presence of thorax indentations. The presence (score 1) and absence (score 0) of thoracic indentations were scored. Student’s t test was used to assess the statistical differences.

**Supplemental references**

1. Verstreken P, Ly CV, Venken KJ, Koh TW, Zhou Y, et al. (2005) Synaptic mitochondria are critical for mobilization of reserve pool vesicles at Drosophila neuromuscular junctions. Neuron 47: 365-378.

2. Morais VA, Verstreken P, Roethig A, Smet J, Snellinx A, et al. (2009) Parkinson's disease mutations in PINK1 result in decreased Complex I activity and deficient synaptic function. EMBO Mol Med 1: 99-111.
